# Clinical data on rare case of spontaneous disappearance of intracranial Aneurysm

**DOI:** 10.1016/j.dib.2020.105874

**Published:** 2020-06-18

**Authors:** Shigeomi Yokoya, Akihiko Hino, Hideki Oka, Naoto Shiomi

**Affiliations:** a Department of Neurosurgery, Saiseikai Shiga Hospital, Imperial Gift Foundation Inc., Ritto, Shiga, Japan

**Keywords:** Anterior circulation, Clinoid segment of internal carotid artery, Unruptured aneurysm, Spontaneous disappearance

## Abstract

The data contained within this paper are related to another paper entitled “Rare Spontaneous disappearance of intracranial aneurysm” (Yokoya et al., 2020). Spontaneous disappearance of an unruptured non-giant aneurysm of the anterior circulation is very rare. We identified a saccular cerebral aneurysm, which disappeared spontaneously and was followed up for 12 years. The present article describes the relevant clinical data of the patient, including her medical history and imaging findings.

Specifications table

**Table:** 

Subject	Clinical Neurology
Specific subject area	Intracranial aneurysm
Type of data	Image
How data were acquired	Medical records of a patient were retrospectively reviewed
Data format	Original and constructed 3-dimensional image of her magnetic resonance angiography
Parameters for data collection	A patient with disappeared intracranial aneurysm
Description of data collection	Retrospectively acquired images of the patient
Data source location	Saiseikai Shiga Hospital, Imperial Gift Foundation Inc., Ritto, Shiga, Japan.
Data accessibility	The raw data (in.jpg format) can be downloaded from the Mendeley Data (DOI: 10.17632/3rsmrpfssy.1)
Related research article	Shigeomi Yokoya, Akihiko Hino, and Hideki Oka Rare Spontaneous disappearance of intracranial aneurysm. World Neurosurgery. (2020) [1]

## Value of the Data

• The data demonstrates a rare case of spontaneous disappearance of an intracranial aneurysm.
• The data will be beneficial to neurosurgeons, neurologists, and patients with intracranial aneurysms.
• The data will provide knowledge regarding rare variations in the natural history of an aneurysm.
• The raw MRI data that were used to generate the MRA data (linked to the Mendeley Data) will be helpful for the audience of the original paper to exclude the possibility that the "disappearance of aneurysm" on MRA was not due to an imaging artifact.

## Data

Figures demonstrating the disappearance of the unruptured non-giant anterior circulation saccular intracranial aneurysm. (A, B) Initial magnetic resonance angiography (MRA) showing a left clinoid segment (C3) saccular aneurysm; Imaging was acquired using Intera 1.5T (Philips Medical Systems, Best, The Netherlands) (FOV:180 mm*180 mm, TR:20 msec, TE 6.9 msec, FA 20 deg.) (C, D) MRA images obtained 12 years after the initial examination showing the disappearance of the aneurysm; Imaging was acquired using Discovery MR750w 3T (GE Healthcare, Milwaukee, USA) (FOV:200 mm*200 mm, TR:25 msec, TE 3.4 msec, FA 18 deg.)

## Experimental design, materials, and methods

A 53-year-old woman underwent magnetic resonance imaging and magnetic resonance angiography (MRA) for the brain. She had a medical history of uterine polyps and an ovarian cyst in her thirties; both conditions needed surgery. She had no family history of intracranial aneurysm or subarachnoid hemorrhage.

**Fig. 1:**
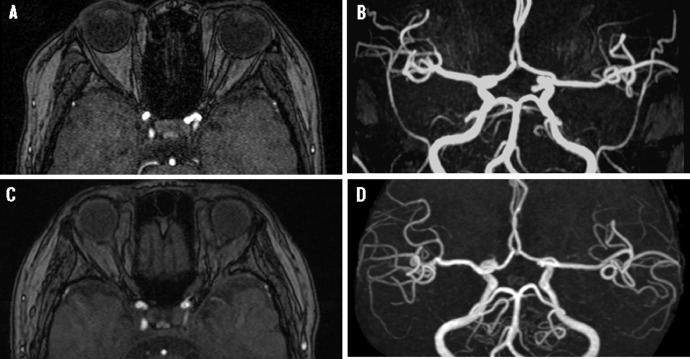

The MRA suggested the presence of an unruptured cerebral aneurysm (Fig. 1A, B). For further examination, she underwent digital subtraction angiography (DSA), which revealed a clinoid segment (C3) aneurysm that had grown into the carotid cave.

An aneurysm was observed and followed up annually using MRA. She quit smoking during the follow-up period, at the age of 55 years, 2 years after the aneurysm was discovered. She started taking medication for diabetes mellitus and hypertension at the age of 64 years. She was obese at the first visit, and the obesity did not improve.

For 11 years, the aneurysm did not change on MRA examination. However, 12 years after the first diagnosis, the aneurysm disappeared on MRA (Fig. 1C, D) and DSA examination.

We retrospectively reviewed her medical record to check her condition, medications, and clinical imaging. Informed consent was obtained from the patient and her family for publication.

## Declaration of Competing Interest

We have no declarations of interest.

## Appendix Supplementary materials

Image, application 1
